# The *Adhesion *GPCR GPR125 is specifically expressed in the choroid plexus and is upregulated following brain injury

**DOI:** 10.1186/1471-2202-9-97

**Published:** 2008-10-03

**Authors:** Chris Pickering, Maria Hägglund, Joanna Szmydynger-Chodobska, Fernanda Marques, Joana A Palha, Linn Waller, Adam Chodobski, Robert Fredriksson, Malin C Lagerström, Helgi B Schiöth

**Affiliations:** 1 Uppsala University, Department of Neuroscience, Functional Pharmacology, BMC, Box 593, SE-75124, Uppsala, Sweden; 2 Uppsala University, Department of Neuroscience, Division of Developmental Genetics, BMC, Box 587, SE-75124, Uppsala, Sweden; 3 The Warren Alpert Medical School of Brown University, Department of Clinical Neurosciences, Aldrich Building 405, 593 Eddy Street, Providence, RI 02903, USA; 4 University of Minho, Life and Health Sciences Research Institute (ICVS), School of Health Sciences, Campus Gualtar, 4710-057, Braga, Portugal

## Abstract

**Background:**

GPR125 belongs to the family of *Adhesion *G protein-coupled receptors (GPCRs). A single copy of GPR125 was found in many vertebrate genomes. We also identified a *Drosophila *sequence, DmCG15744, which shares a common ancestor with the entire Group III of *Adhesio*n GPCRs, and also contains Ig, LRR and HBD domains which were observed in mammalian GPR125.

**Results:**

We found specific expression of GPR125 in cells of the choroid plexus using *in situ *hybridization and protein-specific antibodies and combined *in situ*/immunohistochemistry co-localization using cytokeratin, a marker specific for epithelial cells. Induction of inflammation by LPS did not change GPR125 expression. However, GPR125 expression was transiently increased (almost 2-fold) at 4 h after traumatic brain injury (TBI) followed by a decrease (approximately 4-fold) from 2 days onwards in the choroid plexus as well as increased expression (2-fold) in the hippocampus that was delayed until 1 day after injury.

**Conclusion:**

These findings suggest that GPR125 plays a functional role in choroidal and hippocampal response to injury.

## Background

G protein-coupled receptors (GPCRs) form one of the largest gene families in the human genome and participate in a wide range of physiological and sensory functions. Their ability to mediate specific signals over the cell membrane contributes to the fact that about 25% of the 100 top-selling pharmaceutical targets are GPCRs [1]. However, many of these receptors are still orphans with unknown functions. GPCRs can be classified into five main families; *Glutamate*, *Rhodopsin*, *Adhesion*, *Frizzled/Tas2 *and *Secretin *[2]. The characteristic 7TM domain is the hallmark of all GPCRs and the members within each of the five families are likely to share common descent. These main families can also be distinguished by different types of N-termini. Most of the receptors from the *Glutamate*, *Secretin*, *Frizzled *and the *Adhesion *families have long N-termini with functional domains while very few of the *Rhodopsin *receptors have such domains. The N-termini of the *Adhesion *family, also known as family B2 [3] or LNB-TM7 [4], are very different from those of the other groups since these consist of long stretches rich in serine and threonine which produce a stalk-like formation projecting from the cell membrane. This structure is probably very important for cell-to-cell interactions. Interestingly, these N-termini frequently contain multiple functional domains such as epidermal growth factor (EGF), cadherin, lectin, laminin, olfactomedin, immunoglobulin or trombospondin that are most commonly found in other types of protein such as integrins, cadherins and tyrosine kinases. While the *Rhodopsin *family is by far the largest GPCR family with many well studied amine and peptide binding receptors, the *Adhesion *family is the second largest family with 33 members in the human genome. Several of them were identified recently [5,6] and the functional roles of these are not well understood. The *Adhesion *GPCRs can be further subdivided into groups I-VIII based on phylogeny (sequence similarity) and virtually all of them contain the GPCR proteolytic site (GPS) domain [6].

We previously identified three novel receptors; GPR123, GPR124 and GPR125, which phylogenetically cluster together with the *Adhesion *GPCR family [5]. These receptors form *Adhesion *Group III [6]. GPR123 is highly expressed in cortical layer 5 pyramidal neurons and in most parts of the thalamus and the spinal cord [7]. GPR124 and GPR125 are also known as tumor endothelial marker 5 (TEM5) and TEM5-like, respectively [8]. Members of the tumor endothelial marker family were originally identified by searching for genes with both elevated expression under tumor angiogenesis [9] and cell surface expression to facilitate the drug design process [10]. Originally, TEM5 (GPR124) was classified as GPCR-like while TEM5-like (GPR125) has only been discussed in [8]. Expression of both TEM5 and TEM5-like are elevated in human tumors and proteins for these genes were found to bind the human homologue of the *Drosophila *discs large tumor suppressor gene (hDlg) which localizes receptors to the cell surface [8]. With respect to localization by Northern blot analysis, GPR125 (TEM5-like) was identified throughout many peripheral human tissues with a low expression in the brain but high expression in human brain tumors [8]. A second study suggested a developmental role for GPR125 [11]. GPR125 is found in highly proliferative adult spermatogonial progenitor cells (SPCs) and multipotent adult spermatogonial-derived stem cells (MASCs) but is downregulated after differentiation, thus suggesting GPR125 as a marker for cultivation of SPCs or MASCs which may later be used for transplantation therapies [11].

The purpose of this study was to describe the evolutionary history of GPR125 and map the mRNA expression pattern using *in situ *hybridization and real-time RT-PCR on mouse brain and peripheral tissues. Additionally, we used immunohistochemistry to localize GPR125 to a given cell type and animal models to determine whether expression of this new GPCR changes in response to pathophysiological stimuli.

## Methods

### Sequence retrieval and editing

Full-length *Adhesion*-sequences for human (Hs), mouse (Mm) [6] and chicken (Gg) [12] were downloaded from previously published articles. The sequence for rat (Rn) corresponded to accession number XP_223485.4. The human sequences were truncated to include only the TM regions according to rps-blast at  (conserved domain database, CDD – 12589 PSSMs) and served as probes against *Tetraodon nigroviridis *(Tn), *Takifugu rubripes *(Tr) and *Drosophila melanogaster *(Dm). Tn and Tr were both investigated using BLAST-like alignment tool (BLAT) searches against their genome at . Dm, on the other hand, was searched against its proteome with blastp searches at . To rule out further hits, the previously obtained sequences were used as probes in new BLAT-searches against their respective genomes. The potential sequences were manually assembled in Editseq from the DNA-star package version 5.07 (DNASTAR, Madison, Wisconsin, United States) according to the canonical splice site GT...AG. The assembled sequences were then aligned with their probes in the Windows version of ClustalW 1.83 [13] to confirm consistency of splice sites. The N-terminals of the selected sequences were assembled in the same manner as mentioned above but with the full-length human protein as probes. They were then searched for their domain extent in rps-blast with a cutoff value of 0.1.

### *Adhesion *confirmation

To confirm that the sequences of interest were *Adhesion *GPCR members, an in-house program was applied. The program searched the sequences against a blast database comprised of all human GPCR families (in-house dataset) and additional sequences from the *Methuselah *family [3] and the *cAMP *family [14]. The sequences had to fulfill the following criteria; that the first three hits were indeed *Adhesion *and that an overall of five of the first ten hits were *Adhesion*.

### Phylogenetic analysis

The newly discovered sequences related to GPR125 and their homologs were collected into a FASTA file. The entire human *Adhesion *GPCR repertoire together with Gg and Mm members of group III, according to previous division of the *Adhesion *family conducted by Bjarnadottir and colleagues [6], additional *Methuselah *sequences [3] and the complete human *Secretin *family (in-house data) was also included in the file. Subsequent multiple alignments were performed on the file using the Windows version of ClustalW 1.83 [13] with default parameter settings. Using seqboot from the Win32 version of the PHYLIP 3.65 package [15], the alignment was bootstrapped 1000 times. The output file from seqboot was then subjected to protdist from the Win32 version of the PHYLIP 3.65 package using the Jones-Taylor-Thornton matrix [16]. The neighbour command with default settings, from the Win32 version of the PHYLIP 3.65 package, was then used to construct neighbour-joining trees from the distance file produced by protdist. In order to combine the trees into a consensus tree, the command consense from the Win32 version of the PHYLIP 3.65 package was used with the majority rule and then plotted in the Win32 version of TreeView 1.6.6 [17]. The consensus tree was then weighted in TreePuzzle version 5.2 [18] with the settings; Tree reconstruction, evaluate user-defined trees, exact (slow) parameter estimates, Mueller-Vingron model of substitution, gamma distributed rates as model of heterogeneity estimated from the data set and eight categories of gamma rate. The remaining parameters were kept default.

### Experimental subjects

All experiments were approved by the institutional animal care and use committees in Sweden, Portugal and the United States. Animal care procedures followed the guidelines of Swedish regulation (Animal Welfare Act SFS1998:56) and EU legislation (Convention ETS123 and Directive 86/609/EEC).

### Gene expression using qPCR

#### Tissue collection

To prepare the mouse tissue panel, 4 adult, male Sv129 (Alab, Sollentuna, Sweden) were housed in a climate-controlled facility (12 hr light/dark cycle with lights on at 07.00) with constant temperature (22–23°C) and 55% humidity. Animals were given 7 days to acclimatize to the local conditions and had free access to water and R36 food pellets (Labfor Lactamin, Vadstena, Sweden). Between 3 and 6 hours into the light period, animals were decapitated for dissection of brains and various peripheral tissues. Brains were placed in a brain matrix for dissection into areas indicated in Figure 4 while peripheral organs were taken in their entirety. All samples were frozen on a dry ice block before immersion into RNALater solution (Ambion). Following 1 hr of incubation at room temperature to allow complete penetration of the tissue, samples were stored at -80°C until further processing.

#### RNA extraction and cDNA synthesis

Tissue samples were homogenized in TRIzol reagent (Invitrogen, Sweden) using a Branson sonicator. Total RNA was extracted according to the manufacturer's protocol (Invitrogen, Sweden). To remove contamination, samples were treated with DNase I (Roche Diagnostics, Sweden) at 37°C for 4 hours followed by 15 minutes at 75°C to inactivate the enzyme. To confirm that there was no DNA contamination, a PCR was performed on the RNA sample using reference gene GAPDH as a positive control. If bands were detected after this PCR, DNase I treatment was repeated until no amplification was observed in the RNA. The concentration of RNA was measured with the Nanodrop^® ^ND-1000 spectrophotometer (NanoDrop Technologies, Delaware, USA). cDNA was then synthesized according to the manufacturer's protocol with M-MLV reverse transcriptase (GE Healthcare, Sweden) and random hexamers (GE Healthcare, Sweden). To ensure presence of cDNA in the samples, a PCR was run (again with GAPDH as positive control) but this time a band was expected for each sample.

#### Primer Design

Primers were designed using Beacon Designer v4.0 (Premier Biosoft, USA) and specificity was verified via a BLAST search against the genome. Mouse primers (F = forward, R = reverse) were as follows: GPR125 (F – atg ctt gtg aac ctg tgc ttt c, R – cgc tgg cat ttc tgg tct gg), reference genes (glyceraldehyde-3-phosphate-dehydrogenase (GAPDH), F – gcc ttc cgt gtt cct acc, R – gcc tgc ttc acc acc ttc; β-tubulin, F – agt gct cct ctt cta cag, R – tat ctc cgt ggt aag tgc; ribosomal protein L19, F – aat cgc caa tgc caa ctc, R – gga atg gac agt cac agg; histone H3b, F – cct tgt ggg tct gtt tga g, R – cag ttg gat gtc ctt ggg; cyclophilin, F – ttt ggg aag gtg aaa gaa gg, R – aca gaa gga atg gtt tga tgg; β-actin, F – cct tct tgg gta tgg aat cct gtg, R – cag cac tgt gtt ggc ata gag g; succinate dehydrogenase complex subunit B, F – tgg tgg aac gga gac aag, R – cag cgg tag aca gag aag g).

#### qPCR

Quantitative PCR was performed in 96-well plates with the MyIQ iCycler real-time detection instrument (Bio-Rad, Sundbyberg, Sweden). SYBR Green I (Invitrogen) dissolved in Tris-EDTA (pH 8) was used as the fluorescent reporter. The 20 μl reaction volume consisted of 2 μl 10× PCR buffer, 20 mM dNTP, 50 mM MgCl_2_, 0.05 μl forward and reverse primer (100 pmol/μl), 1 μl DMSO, 1:50000 SYBR Green I, 0.08 μl (5 units/μl) Taq DNA polymerase (Biotools, Spain) and 9.52 μl RNase-free water mixed with 5 μl (5 ng/μl) of cDNA template. For each primer pair, 50 PCR cycles were run with parameters of 95°C for 15 s, 58.2–62°C (depending on primer) annealing for 30 s followed by extension at 72°C for 30 s. A melting curve analysis was performed following the cycles to ensure that a single PCR product was formed in the reaction. Primers were originally optimized by running a temperature gradient (annealing temperature from 52°C to 62°C across the rows of the plate) to give an optimal temperature of 62°C for mouse GPR125.

#### Normalization and data analysis

Data was obtained using the MyIQ software (Bio-Rad) and a normalization factor was calculated using the 7 reference genes and the GeNorm method [19]. Primer efficiency was calculated using the LinRegPCR method and corrected Ct values were calculated using the assumption-free analysis method of [20]. Expression in the tissue with the smallest value was set to 1 and all other values were expressed relative to this minimum. Samples were run in duplicate and repeated if necessary.

### Gene expression using free floating tissue section *in situ *hybridization

#### Tissue collection and sectioning

Adult, male Sv129 mice were anaesthetized with a 1:1 mixture of Dormitor (70 μg/g, Orion) and Ketalar (7 μg/g, Pfizer) and transcardially perfused with PBS followed by 4% formaldehyde. Brains were dissected and then fixed overnight in 4% formaldehyde. After washing in PBS, brains were embedded in 4% agarose and sectioned (70 μm) on a Leica vibratome. Slices were then dehydrated through a series of methanol washes and stored in 100% methanol at -20°C until use.

#### Synthesis of riboprobes

Antisense and sense probes for GPR125 were synthesized from the BMAP clone ID# 5718990 (Invitrogen). DNA was prepared with the JETstar 2.0 Plasmid Purification Midi Kit/50 (Genomed, Germany) and the clone was then sequenced at MWG  to verify the accuracy of the clone. The antisense probe was synthesized with T3 RNA polymerase and restriction enzyme SpeI (Fermentas, Sweden) while the sense probe was synthesized using T7 RNA polymerase after restriction with AflII (New England Biolabs, Sweden). Both types of probes were labeled with digoxigenin-11-UTP (Roche Diagnostics, Sweden). Probe concentration was measured with the Nanodrop^® ^ND-1000 spectrophotometer (NanoDrop Technologies, Delaware, USA) before storage at -80°C.

#### In situ hybridization

Sections were re-hydrated through a series of methanol solutions (75%, 50%, 25%) dissolved in PBT (phosphate-buffered saline (PBS) with 0.1% Tween-20 (Sigma-Aldrich)). Sections were bleached in 6% hydrogen peroxide, permeated with 0.5% Triton X-100 (Sigma-Aldrich), digested in proteinase K (20 μg/ml; Invitrogen) and post-fixed in 4% formaldehyde with PBT washes between the steps. Sections were pre-hybridized at 55°C in hybridization buffer (50% formamide, 5× SSC (pH 4.5), 1% SDS, 50 μg/ml tRNA and heparin (both Sigma-Aldrich) and 0.1% DEPC-treated water. DIG-labeled probes were heat denatured at 80°C for 5 min before cooling on ice for 5 min. For GPR125, 400 ng probe/ml was added to each section and hybridization was performed overnight at 55°C. Unbound probe was washed using a series of washing steps of 3 × 30 min each buffer. The first wash buffer (50% formamide, 2× SSC (pH 4.5), 0.1% Tween-20 in DEPC-treated water) was followed by a second (50% formamide, 0.2× SSC (pH 4.5), 0.1% Tween-20 in DEPC-treated water). Sections were then washed in Tris-buffered saline with 0.1% Tween-20 (TBST) followed by a 2 hr incubation in blocking solution (Roche Diagnostics, Sweden). Anti-DIG antibody conjugated to alkaline phosphatase (Roche Diagnostics, Sweden) was diluted 1:5000 in blocking solution and sections were incubated at 4°C overnight. Sections were washed 5 × 10 min in TBST with 2 nM levamisol (GTF Fisher, Sweden) to block endogenous alkaline phosphatase activity followed by 10 min in NTMT (100 mM NaCl, 10 mM Tris-HCl (pH 9.5), 50 mM MgCl_2_, 0.1% Tween-20) with 2 nM levamisol. Sections were then developed at 37°C using the BM Purple-AP enzyme substrate (Roche Diagnostics, Sweden).

#### Image acquisition

Sections were mounted in 50% glycerol and photographed with a Leica MZ16F microscope (Leica Microsystems, Germany), Leica DFC300 FX camera and FireCam software.

### Co-localization using combined *in situ *hybridization/immunohistochemistry

#### Antibodies

Primary antibody dilutions were as follows: 1:400 mouse anti-pan cytokeratin (Sigma-Aldrich), 1:500 chicken anti-GFAP (Chemicon) and 1:1000 for nucleic acid stain 4,6-diamidino-2-phenylindole (DAPI) (Roche Diagnostics, Sweden). Secondary antibodies were diluted as follows (all from Invitrogen): 1:800 anti-Mouse-Alexa 488, and 1:200 anti-Chicken-Alexa 647.

#### Modifications to the in situ protocol

Primary antibodies were incubated at 4°C overnight simultaneously with the anti-DIG antibody. Sections were developed using the red fluorescent enzyme substrate Fast Red according to the manufacturer's instructions (Roche Diagnostics, Sweden). Slices were stained for 5 minutes in 1:1000 diluted 4,6-diamidino-2-phenylindole (DAPI; Roche Diagnostics, Sweden) in DEPC-treated water and then placed on Superfrost Plus glass slides (Menzel-Glaser, Germany) and protected from light. Secondary antibodies were added to the glass and incubated for 2 hours followed by mounting with coverslip and the antifade DTG mounting medium (2.5% DABCO (Sigma-Aldrich D-2522), 50 mM Tris pH 8.6, 90% glycerol).

#### Image acquisition

Slides were photographed using the Zeiss Laser Scanning Microscope LSM510 confocal microscope system version 4.2 and Zeiss LSM Image Examiner software. All 4 fluorophores were acquired together via customization of track settings and filter/mirror positions and channels were overlayed by the software. When necessary, the z-stack function was used to acquire images of several layers of the tissue which was rendered 3-dimensionally by the software to aid in visualization of the co-localization.

### Immunohistochemistry using GPR125 antibody

The rabbit polyclonal antibody against GPR125 was obtained from the Swedish Human Proteomics Resource project [21]. Two naïve Wistar rats were sacrificed at 3 weeks of age by decapitation and the brain was dissected and rapidly frozen in isopentane at -22°C followed by storage at -80°C until sectioning. Sections of 25 μm were made using a cryostat, thaw-mounted onto SuperFrost Plus slides (Menzel-Glaser, Germany) and stored at -20°C until analysis. Sections were re-hydrated in PBS and fixed in methanol at -20°C followed by treatment with 0.1% Triton X-100. Sections were then incubated in blocking solution (0.45% fish skin gelatin (Sigma-Aldrich)) for 1.5 hr and incubated overnight with primary antibodies (anti-GPR125 in rabbit at 1:100; anti-cytokeratin in mouse at 1:400 (Sigma); anti-MCT-2 in chicken at 1:250 (Chemicon)). After PBS wash, sections were incubated with secondary antibodies (anti-rabbit-Alexa 488 at 1:500; anti-mouse-Alexa 546 at 1:500 or anti-rabbit-Alexa 546 at 1:500; anti-chicken-Alexa 488 at 1:500; all from Invitrogen). Finally, DAPI nuclear stain (Sigma-Aldrich) was added and sections were mounted in DTG medium as described previously.

### Induction of inflammatory response by LPS

Inflammation was induced by intraperitonial injection of lipopolysacharide (LPS) as described previously [22]. Briefly, animals were injected i. p. with 5 μg/g body weight LPS (*Escherichia coli *serotype O55:B5, Sigma-Aldrich) or saline (0.9% NaCl). At 1, 3, 6, 12, 24 and 72 hours after injection, the choroid plexus was rapidly removed and stored at -80°C until further processing. A minimum of five pools of choroid plexus from n = 4 animals were prepared and analyzed for each time point.

### The rat traumatic brain injury (TBI) model

Adult male Long-Evans rats weighing 280–320 g were purchased from Harlan Sprague-Dawley (Indianapolis, USA). The controlled cortical impact model of TBI was used as previously described [23]. Rats were anesthetized with pentobarbital sodium (60 mg/kg, i. p.) and were placed in a stereotactic frame. A 4-mm craniotomy was performed on the right side of the skull to expose the dura, with the center of the opening located 3 mm posterior to bregma and 2.5 mm lateral to the midline. The velocity of impact was 5 m/sec and the duration of impact was 50 msec. The diameter of impactor's tip was 2.5 mm and the depth of brain deformation was set at 3 mm. Immediately after the insult, the scalp was closed with a nylon suture and the rats were allowed to recover in their cages. At 2, 4, 6, 24, 48 and 96 h after injury (n = 3–6 animals per time point), samples of the lateral ventricle choroid plexus and the hippocampus ipsilateral and contralateral to the injury side were collected for qPCR analysis. qPCR results are presented as the mean number of copies of GPR125 mRNA per 100 copies of cyclophilin A mRNA ± S.E.M. Results were analyzed by ANOVA followed by the Neuman-Keuls test for multiple comparisons. Comparisons in GPR125 expression were made for ipsilateral vs contralateral tissues and over time within the ipsilateral and contralateral sides.

## Results

### Sequence retrieval and phylogenetic analysis

**Figure 1:**
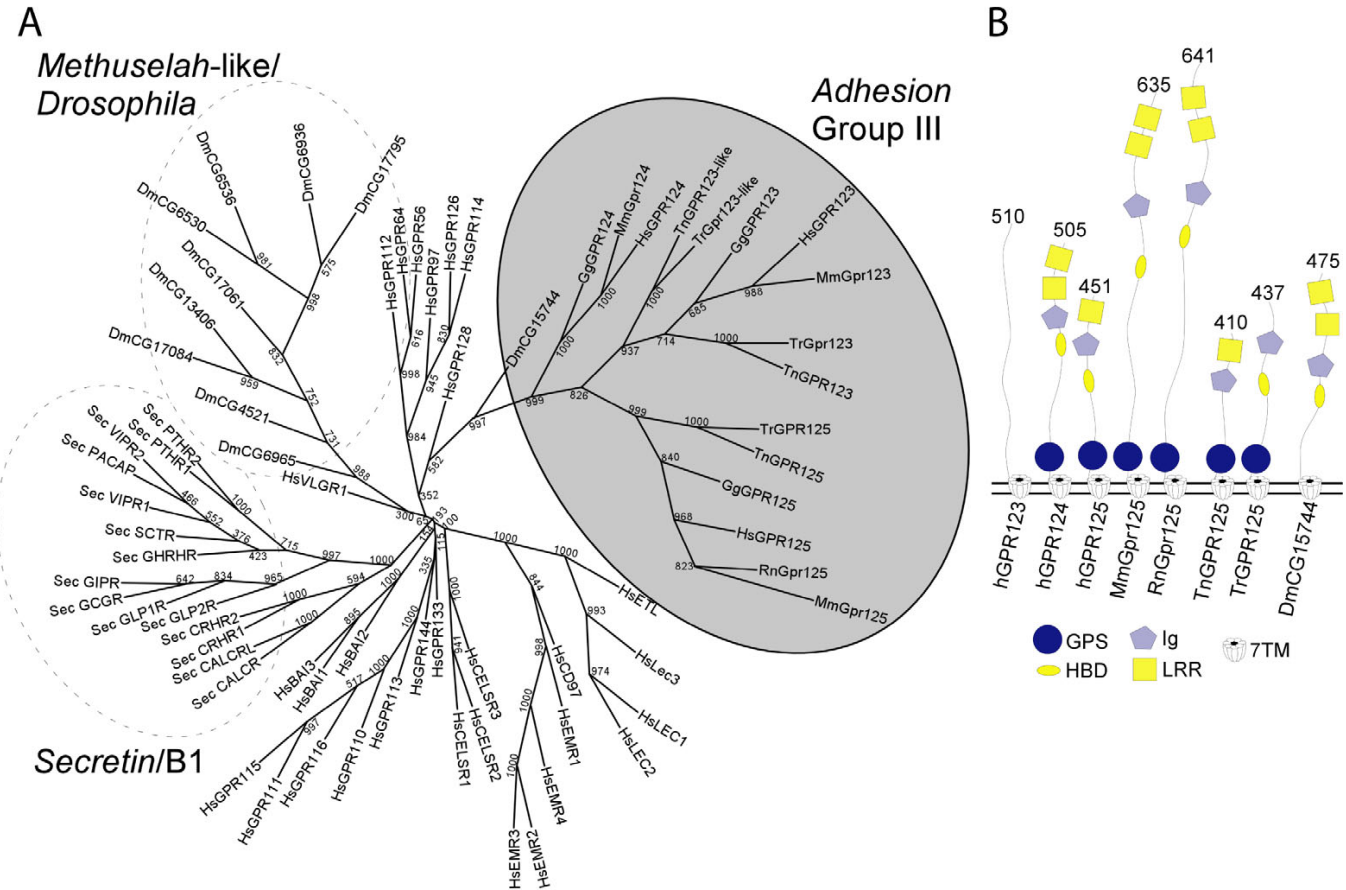
**A) A consensus neighbour-joining tree calculated in protdist and neighbour from the Phylip package version 3.66 with default settings and 1000 replicates. ***Methuselah*-like/*Drosophila *and *Secretin*/B1 sequences were downloaded from (Harmar, 2001). The *Adhesion *group has been named III according to Bjarnadottir and colleagues (Bjarnadottir et al., 2004) and is indicated by the shaded circle. B) Representation of *Adhesion *Group III N-terminals and their incorporated domains identified via rps-blast at  with a cutoff value of 0.1. The sequences are grouped according to Bjarnadottir and colleagues division in group I-VIII. Represented sequences are H – *Homo sapiens*, Tn – *Tetraodon nigroviridis*, Tr – *Takifugu rubripes *and Dm – *Drosophila melanogaster*. Possible domains are GPS – GPCR proteolytic site, HBD – hormone binding domain, LRR – leucine rich repeats, Ig – immunoglobulin and 7TM – seven transmembrane domains. The numbers above the N-terminal indicate the length in number of amino acids.

A phylogenetic tree was prepared using the *Adhesion*, *Secretin*, and *Methuselah *families (Figure 1A). GPR123, GPR124 and GPR125 grouped together (shaded grey in Figure 1A) and the *Drosophila *receptor DmCG15744 branches before the cluster of these three receptors. Domains were identified through the conserved domain database (rps-blast) and the N-terminal regions with each domain are illustrated in Figure 1B. The GPCR proteolytic site (GPS) domain is conserved in all sequences except DmCG15744. Rodent GPR125 have longer N-terminal regions than human GPR125 but all contain hormone binding domains (HBD), immunoglobulin (Ig) and leucine-rich-repeat (LRR) domains. In contrast, teleosts either have an HBD and Ig domain (*Takifugu rubripes*) or Ig and LRR domain (*Tetraodon nigroviridis*) but not all three. DmCG15744, however, contained all three domains and was therefore consistent with rodent GPR125. An analysis of the conservation of intron-exon boundaries is provided in Additional file 1. Intron-exon boundaries were conserved between human, rat and mouse GPR125 and between the two teleost sequences but there was no conservation of boundaries between these and DmCG15744.

GPR125 was present in the teleosts (*Tetraodon nigroviridis *(Tn) and *Takifugu rubripes *(Tr)) as well as the chicken and mammals. The full alignment of these sequences is presented in Additional file 2. Amino acid similarity in the transmembrane regions was high between human and mouse (94.3%) and also human and chicken (85.2%) and human versus the teleosts (Tn 68.5% and Tr 66.8%). DmCG15744 had a 17.8% amino acid similarity with the human GPR125 and similarity to Tn and Tr was 31.3% and 28.5% respectively.

### GPR125 expression via qPCR

**Figure 2:**
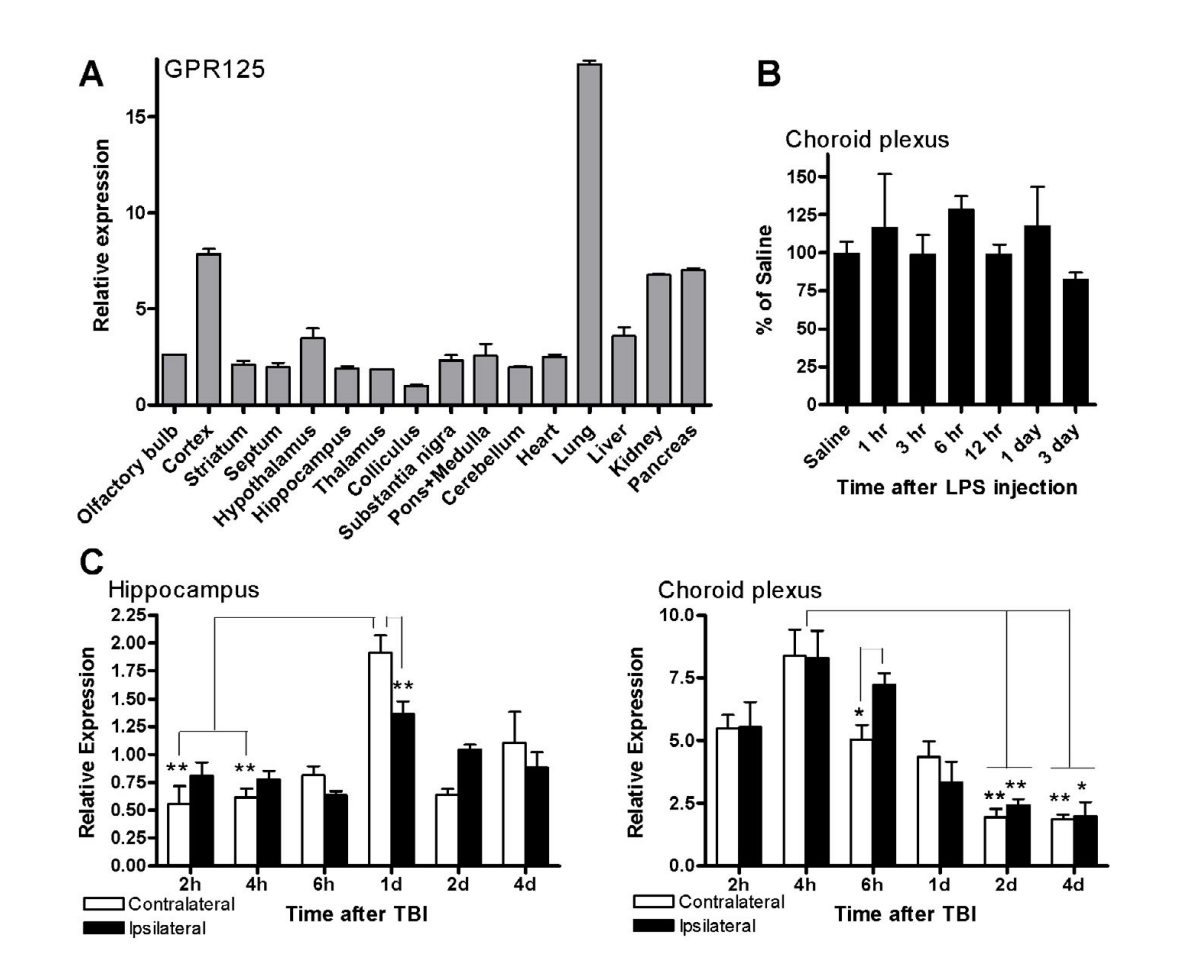
**A) Localization of GPR125 using qPCR in mouse brain and peripheral tissues.** Values are expressed relative to the minimum expression value for this analysis (colliculus). B) GPR125 expression was not changed following induction of an inflammatory response via LPS injection. C) GPR125 expression was affected by TBI. GPR125 in the choroid plexus was upregulated at 4 h after injury while the upregulation in the hippocampus did not occur until 1 day post-TBI, suggesting a role of the CSF pathways in post-traumatic regulation of GPR125 expression in the choroid plexus and hippocampus.

To obtain an overview of the expression of mRNA for GPR125, qPCR was performed on a panel of mouse brain regions and peripheral organs (Figure 2A). To simplify presentation, values were normalized to the tissue with minimum expression of GPR125 (colliculus = 1). Expression in most brain tissues was approximately 2-fold that of the colliculus such that no differential pattern of expression was observed between striatum, hippocampus or thalamus. In particular, cortical expression of GPR125 was nearly 8-fold higher than that of the colliculus while hypothalamic expression was roughly 3.5-fold higher. Expression in the periphery was higher compared to the brain (numbers in parentheses denote fold-values with respect to colliculus); heart (2.5), liver (3.6), kidney (6.8) and pancreas (7). Highest overall expression for GPR125 was observed in the lungs (18-fold that of colliculus).

### GPR125 expression via *in situ *hybridization

**Figure 3:**
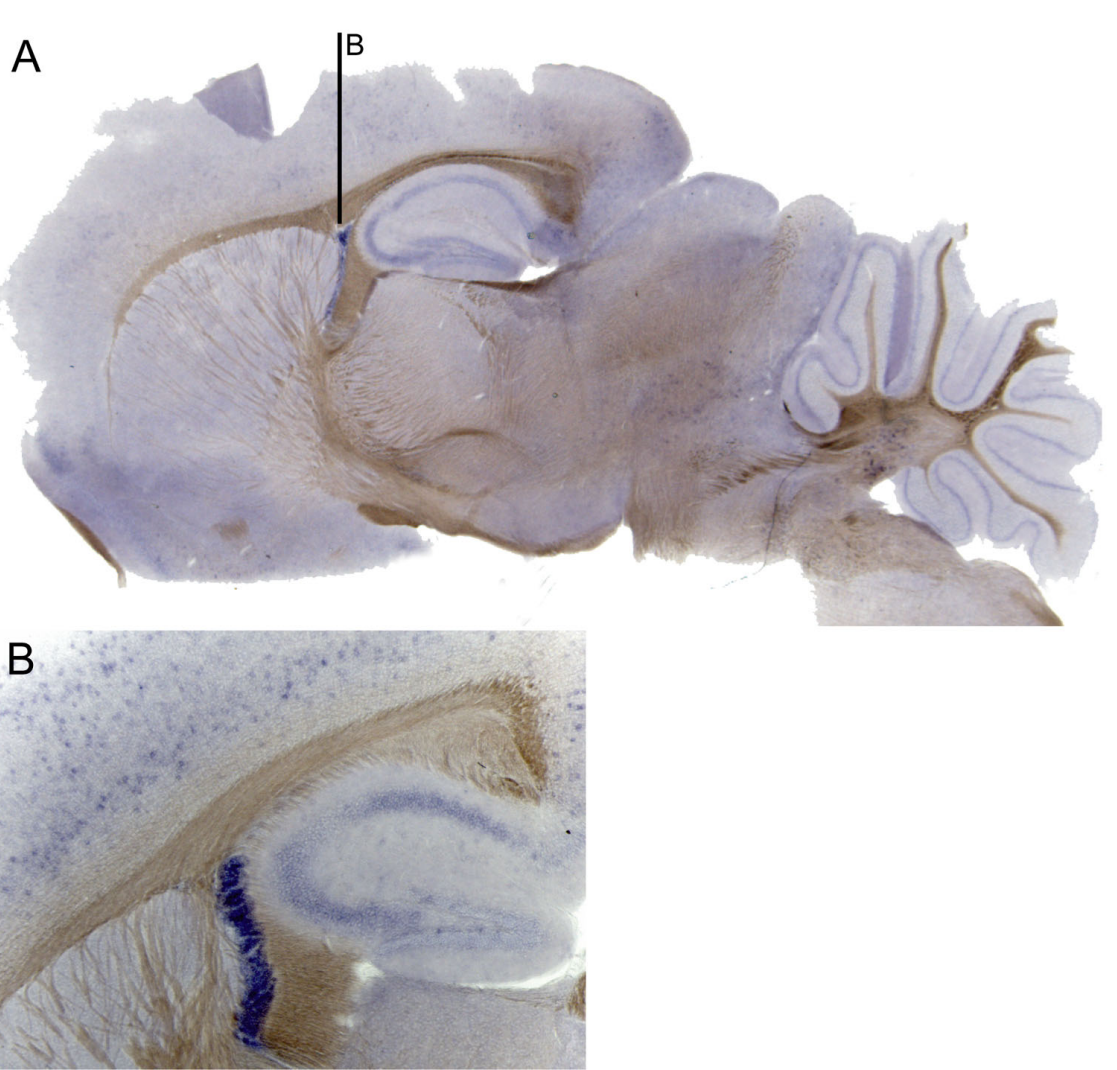
***In situ *hybridization staining on free floating sections using 400 ng of digoxigenin (DIG)-labeled mouse GPR125 antisense probe (A and B) and sense probe (C) as control with the enzyme substrate BM-Purple.** Sagittal section in A and coronal sections in B and C.

For localization, *in situ *hybridization was performed using the visible enzyme substrate BM-Purple and 400 ng of digoxigenin-labeled GPR125 probe. The complete series of this can be seen in Additional file 3. Staining was limited to two key areas; the choroid plexus and neighbouring cerebral cortex (Figure 3A and 3B) and the piriform cortex area 2 (Additional file 3) while no staining was observed in the hippocampus. A sense probe for GPR125 did not stain these areas (data not shown).

### Cellular localization of GPR125

**Figure 4:**
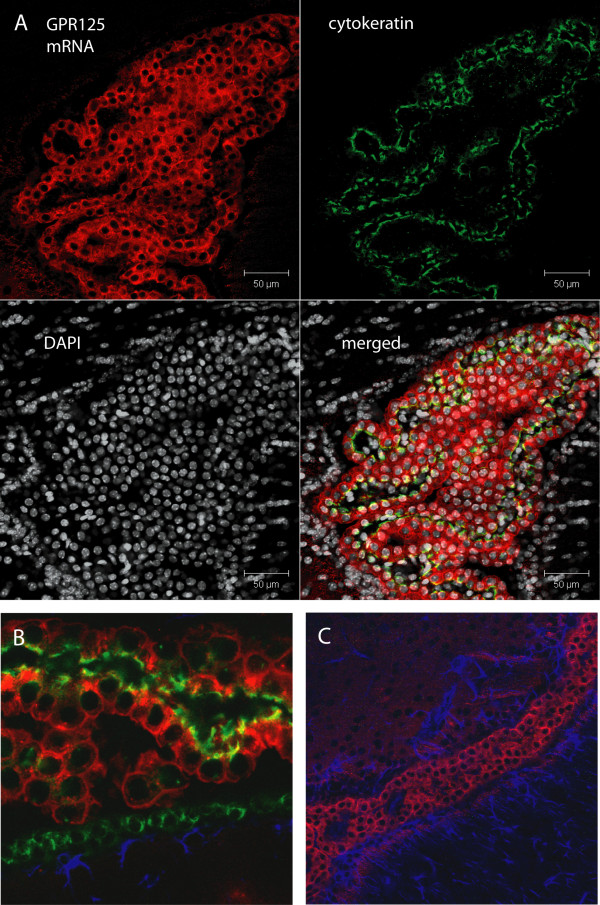
***In situ *hybridization combined with immunohistochemistry on free floating sections using 400 ng of digoxigenin (DIG)-labeled mouse GPR125 antisense probe stained with Fast Red enzyme substrate (A-C), white cell nucleus staining with (1:1000) DAPI (A), green staining with the (1:400) epithelial-cell-specific pan-cytokeratin antibody (A and B) and dark blue (1:500) glial fibrillary acidic protein (GFAP) staining (B and C)**. A) The z-stack function of the confocal microscope rendered 9 layers together to form this 3-dimensional image illustrating GPR125 expression in the cytoplasm around the DAPI-stained nuclei. Separate images are provided for the 3 channels followed by the merged images in the final panel. B) At higher magnification (63× objective), staining for the cytokeratin protein follows the edge of GPR125-labeled cells which illustrates co-localization. C) To show that GPR125 staining is predominantly in the choroid plexus, GFAP was used to stain ependymal cells which line the walls of the ventricle.

To co-localize GPR125 expression to a given cell type, a combined *in situ *hybridization/immunohistochemistry protocol was used. GPR125 was labeled with the fluorescent enzyme substrate Fast Red and could be localized to the cytoplasm around the cell nuclei labeled with DAPI (indicated in white in Figure 4). GPR125 was co-localized to cells of the choroid plexus (Figure 4A and 4B) using the epithelial-cell-specific antibody pan-cytokeratin (green). An enlargement using a 63× objective (Figure 4B) clearly indicates co-localization of GPR125 in choroid plexus cells. Localization of GPR125-positive staining in the choroid plexus itself was confirmed using an antibody against glial fibrillary acidic protein (GFAP, dark blue) which stains GFAP-positive ependymal cells that line the ventricle walls (Figure 4C).

**Figure 5:**
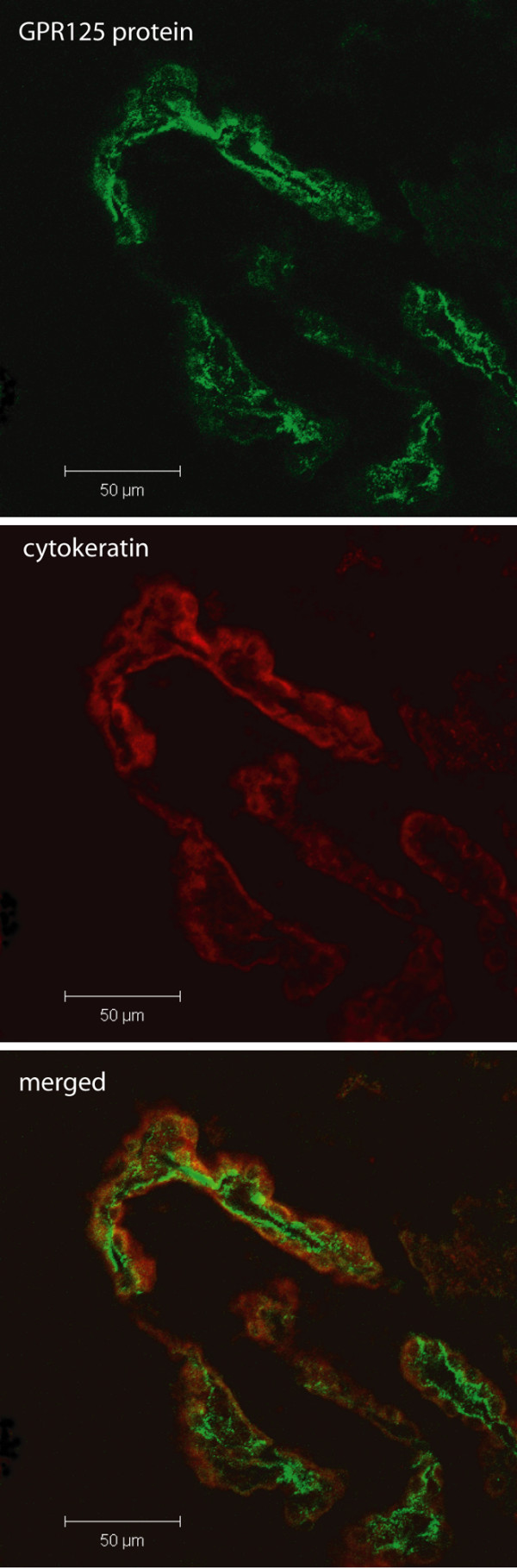
**Immunohistochemistry using an antibody directed against the GPR125 protein (green).** GPR125 was again localized to the choroid plexus cells using the epithelial cell-specific marker cytokeratin (red). This specific staining was observed in sections from the brain of a 3 week old rat, thus confirming expression of GPR125 in pre-adolescence.

To demonstrate localization of the GPR125 protein in the choroid plexus, sections were labeled with the GPR125 antibody (Figure 5). Using the epithelial cell-specific cytokeratin antibody and the z-stack function of the confocal microscope, GPR125 staining (green) was observed in the central portion of the choroid plexus with some overlap with the cytokeratin staining (red) which marks the barrier region of the choroid plexus (Figure 5, merged). Localization of this in the choroid plexus from a 3-week-old animal also indicates central nervous system expression in pre-adolescence. GPR125 did not, however, co-localize with the cerebral vasculature (Additional file 4) as illustrated by double staining with the monocarboxylate transporter MCT-2 which labels the cerebral vasculature and the walls of the ventricle [24].

### Expression of GPR125 after induction of inflammation

To determine whether GPR125 may have an immune function, inflammation was induced by injection of LPS and choroid plexus samples were collected at various times after injection. Expression of GPR125, as measured by qPCR, is illustrated in Figure 2B. No differences were observed (1-way ANOVA; F = 0.63, p = 0.71), thus indicating that induction of inflammatory response does not change GPR125 mRNA expression in the choroid plexus.

### Expression of GPR125 after TBI

To determine whether GPR125 expression changes in response to brain injury, GPR125 mRNA in the choroid plexus and hippocampus was measured after TBI (Figure 2C). GPR125 expression in the choroid plexus significantly changed after injury (ANOVA; F = 22.1, p < 0.001). At 4 h post-TBI, the level of expression of GPR125 in both the ipsilateral and contralateral plexuses was higher than those observed at 2 and 4 days (p < 0.01–0.05) post-TBI. Furthermore, the expression of GPR125 in the ipsilateral choroid plexus at 6 h after injury was elevated compared to the levels found in both the contralateral and ipsilateral plexuses at 2 (p < 0.01) and 4 days (p < 0.05) post-TBI. In addition, a significant difference (p < 0.05) in GPR125 mRNA between the ipsilateral and contralateral plexuses was observed at 6 h post-TBI. GPR125 expression in the hippocampus was also significantly affected by injury (ANOVA; F = 13.7, p < 0.001). The expression of GPR125 in the contralateral hippocampus at 1 day post-TBI was increased compared to the expression levels observed in both the contralateral and ipsilateral hippocampi at 2 (p < 0.01) and 4 h (p < 0.01) after injury. Additionally, a significant difference (p < 0.01) in GPR125 expression between the ipsilateral and contralateral hippocampi was observed at 1 day post-TBI. Overall, GPR125 expression transiently increased in the choroid plexus at 4 h post-TBI followed by a delayed transient increase in the hippocampus at 1 day post-TBI.

## Discussion

GPR125 is evolutionarily conserved in vertebrate genomes. We found this gene to be present in fish, the two teleosts (*Tetraodon nigroviridis *and *Takifugu rubripes*), as well as chicken, suggesting that this gene appeared at least 450 million years ago. It is, therefore, likely to be present in most vertebrates. Moreover, all the vertebrate species we investigated had a single copy of a GPR125 gene without any duplicates. This is in contrast to GPR123 which is present in fish together with an extra copy termed GPR123-like [7] and some other *Adhesion *GPCRs that show differences in gene repertoire between mammals [25]. GPR124, however, does not seem to be present in teleosts while it is found in chicken. Interestingly, our evolutionary mining and phylogenetic analysis identified a *Drosophila *sequence, DmCG15744, which is a common ancestor for the entire Group III of *Adhesio*n GPCRs. DmCG15744 did not group with other *Drosophila *sequences, other *Adhesion *GPCRs or the Methuselah-like GPCRs found in *Drosophila *[3], further supporting the notion that this is an ancestral gene to Group III. DmCG15744 had approximately 20% sequence identity to GPR123, GPR124 and GPR125 but has few introns and, thus, less complex genomic structure than its descents in fish and mammals (see Additional file 2). The fish genes have lower genomic complexity which suggests an increase over evolution in the number of introns in this lineage which concurs with the hypothesis that intron numbers in GPCRs are increasing through the evolution [26]. Interestingly, DmCG15744 shares Ig, LRR and HBD domains with the fish and mammalian GPR125 as well as the mammalian GPR124 (see Figure 1B), providing further strong support for the suggestion that this is a common ancestor to this group. A 17.8% amino acid sequence similarity was observed to human GPR125 and both genes contain HBD, Ig and two LRR domains in the N-terminal regions. However, DmCG15744 does not contain a GPS domain which is surprising as the GPS domain is likely to be found in common ancestors to all the *Adhesion *GPCRs [27]. In addition to this, we performed a second analysis using the Amphioxus sequence published from our recent report [28] and this branch coincided with DmCG15744, suggesting similar conclusions with respect to ancestry and conservation.

The study on a wide tissue panel shows that the mRNA for GPR125 was expressed in the periphery (particularly in the lung, kidney and pancreas) but also in the brain which is in line with previous studies by Yamamoto and colleagues [8]. Our more detailed mapping of the brain found highly specific expression of GPR125 in cells of the choroid plexus in the mouse brain. The choroid plexus is the major source of cerebrospinal fluid (CSF) and is a site of the blood-CSF barrier [29]. Choroid plexus cells are of epithelial origin [29] and were therefore immunopositive for cytokeratin which is a protein specific for epithelial cells [30]. In the choroid plexus, the pattern of staining for GPR125 was similar to that found for cytokeratin staining and its filamentous appearance suggests a role of this receptor in cell-cell adhesion or choroid plexus barrier function. Immunostaining for GPR125 in 3-week-old rats also confirms the expression of this receptor in the pre-adolescence period and suggests a functional role in postnatal development in addition to the previously reported early developmental properties [11]. Interestingly, a choroid plexus and cerebrospinal fluid is present in all vertebrate species but not Amphioxus [31]. Therefore, selective localization of GPR125 in the choroid plexus concurs with our evolutionary findings and suggests a conserved function important for vertebrates.

With knowledge of the evolutionary history and localization of GPR125, it is possible to search for the function of this GPCR. The search for *Adhesion *receptor function is difficult and the function of very few of these receptors have been determined compared to other GPCR classes [32]. Brain-specific angiogenesis inhibitor I (BAI1) is one *Adhesion *receptor of known function which recognizes phosphatidylserine on apoptotic cells and subsequently promotes engulfment of these cells [33]. The involvement of BAI1 in tumor growth [34] and the report of elevated GPR125 in human tumors [8] suggests one line of research to decipher the function of GPR125. Consultation of protein-protein interaction databases (for example, STRING; ) suggests interaction of GPR125 with synapse-associated protein 97 (SAP-97 or hDlg) which was previously reported in [8] and latrophilin, a protein involved in synaptic function and carbohydrate binding [35]. We observed a hormone binding domain in GPR125 but it is difficult to speculate which hormone may bind to this. However, this does suggest a role of GPR125 in the transfer of a message from the periphery via some circulating hormone, thus forming the basis of our study of inflammation and response to brain injury.

Very few genes are expressed selectively in specific brain structures [36] or brain cell types, but our results show abundant and specific expression in cells of the choroid plexus as determined by *in situ *hybridization and immunohistochemistry. Although we also found some expression in the cortex and periphery, it is likely that GPR125 may serve as a specific marker for choroid plexus cells and may thus be useful for the specific targeting of choroid plexus function in transgenic animals. We have previously described the selective production of transthyretin in the choroid plexus and this protein binds the β-amyloid peptide which is known to accumulate during Alzheimer's disease [37]. The selective localization of GPR125 to the choroid plexus may also suggest a role for this in the etiology of Alzheimer's.

The choroid plexus expresses a wide range of receptors and neuropeptides capable of signaling and immunological defense [29] and we hypothesized that GPR125 could be involved in one of these processes. We have previously shown that induction of an inflammatory response by peripheral injection of LPS increases mRNA expression of cytokines IL-1β and TNF-α in the choroid plexus [22]. Inflammation itself induces expression of ICAM-1 [38] and this adhesion molecule is thought to mediate influx of T-cells from the blood to the brain [39,40]. However, induction of inflammation by LPS did not change GPR125 expression at any of the time points investigated, suggesting a lack of GPR125 regulation by changes in peripheral inflammatory mediators. Despite these findings, GPR125 could be involved in signal transduction of peripheral inflammation to the brain through the choroid plexus via mechanisms not requiring alterations in gene expression.

Function of the choroid plexus declines with age [29] and these differences are even more pronounced in neurodegenerative diseases. For example, patients with Alzheimer's disease have a 22% greater choroid plexus epithelial cell atrophy compared to controls [41] and the decrease in cerebrospinal fluid (CSF) production with age [29] may lead to the accumulation of β-amyloid peptides in the brain which could eventually form plaques [42]. Additionally, cerebral ischemia produced by middle cerebral artery occlusion induces apoptotic cell death in the choroid plexus which spreads to the neighbouring hippocampus [43]. Changes in GPR125 expression were measured after traumatic brain injury (Figure 2C). Whereas no differences between the contralateral and ipsilateral expression (except at 6 h post-TBI) were observed in the choroid plexus, the expression transiently increased at 4 h after injury followed by a decrease from 2 days onwards. Increased expression in the hippocampus, a brain structure having a close contact with CSF, was delayed until 1 day after injury. These observations suggest that the CSF pathways may play a role in the injury-mediated regulation of GPR125 expression in both the choroid plexus and hippocampus [44]. These results illustrate an important difference in sensitivity between *in situ *hybridization and qPCR since the hippocampus was positive for mRNA of GPR125 even though expression was considerably less than the cortex. Nonetheless, TBI caused a significant induction of GPR125 mRNA in the hippocampus after initial increases in the choroid plexus (2-fold increase over basal levels). This suggests that despite low basal levels of GPR125 expression in many brain regions, the gene can be upregulated in response to challenges such as brain injury.

Real-time RT-PCR data identified mRNA for GPR125 in small quantities throughout the brain. This was confirmed by *in situ *hybridization data as scattered expression across the cortex (Figure 3B). The expression pattern of GPR125 is very different compared to GPR123 [7] despite the similarity of these receptors and classification together as Group III *Adhesions*. GPR125 is expressed in select regions of the brain and more widely in the peripheral organs while GPR123 was selectively expressed in the brain [7]. A difference in function is also suggested by the presence of HBD, Ig and GPS domains on GPR125 and the absence of these in GPR123 (Figure 1B). The expression pattern of GPR125, in human tissues corresponds somewhat to the expression pattern observed in mouse [8]. Major differences include a lower expression in the human brain and virtually no expression in the human lung [8]. However, it is difficult to compare and quantify differences between Northern blot measurements and qPCR results. Importantly, GPR125 is highly upregulated in tumor samples from human brains [8], which also suggests a role for GPR125 in cancer or an upregulation of this in relation to cancer progression.

## Conclusion

In summary, GPR125 is an *Adhesion *GPCR which has a long evolutionary history in vertebrates. In contrast to GPR123 which is selectively expressed in the brain, GPR125 is expressed both in the brain and periphery. We have demonstrated that GPR125 expression is increased after TBI whereas no changes were observed after peripheral induction of inflammation. The connection of GPR125 expression to brain injury suggests a functional role of this receptor in response to and/or recovery from injury.

## Authors' contributions

CP and MH performed the *in situ*, immunohistochemistry and tissue panel PCR. FM and JAP designed and performed the LPS experiment. JSC and AC designed and performed the TBI experiment. LW, MCL and RF studied the evolutionary aspects of GPR125 with bioinformatics. CP, MCL and HBS prepared the manuscript with input/results from the remaining authors. All authors read and approved the final manuscript.

## Supplementary Material

Additional file 1 **Genomic structure of selected *Adhesion *GPCRs from human (Hs), mouse (Mm), rat (Rn), tetraodon (Tn), fugu (Tr) and drosophila (Dm) where exons are indicated by boxes and introns as lines.** Domains were identified through the conserved domain database (rps-blast) against CDD -12589PSSMs with threshold value 0.1, and are depicted with different colours; Gal_Lectin (yellow), leucine-rich-repeats (green), immunoglobulins (purple), hormR – hormone binding domain (light blue), GPS – G protein-coupled receptor proteolytic site (blue), 7tm – transmembrane domain (red). Immunoglobulins consist of the domains IG and IGcam whereas leucine-rich-repeats consist of LRRCT, LRR_RI, LRR_TYP and COG4886. Exon-phases are displayed with 0 for zeroth phase, + for first phase and ++ for second phase. Interruptions in the sequence are indicated by //// for truncated intron sequence. The sequences have been aligned according to the second exon of the 7TM (see vertical line) since this location is present in all sequences. Click here for file

Additional file 2 **Alignment from ClustalW 1.83 of human members of *Adhesion *Group III together with Gpr125-sequences from *Mus musculus, Rattus norvegicus, Tetraodon nigroviridis, Takifugu rubripes *and *Drosophila melanogaster*.** The alignment has been edited in Jalview 2.2.1 and coloured according to percentage identity where darker colours indicate higher percentage identity. Click here for file

Additional file 3 ***In situ *hybridization panel of GPR125 expression**. *In situ *hybridization panel of GPR125 expression in the mouse brain on free floating sections using 400 ng of digoxigenin (DIG)-labeled mouse GPR125 antisense probe on coronal sections using BM-purple visible enzyme substrate. Click here for file

Additional file 4 **GPR125 co-localization with vasculature.** Labelling of the GPR125 protein (red) did not co-localize with monocarboxylate transporter MCT-2 (green) which is transporter found in the cerebral vasculature and the walls of the ventricle. Click here for file
