# Supplementary figures and images for: The Adhesion GPCR GPR125 is specifically expressed in the choroid plexus and is upregulated following brain injury
Source: BMC Neurosci. 2008 Oct 3;9:97. doi: 10.1186/1471-2202-9-97 (PMC2571103; doi:10.1186/1471-2202-9-97)

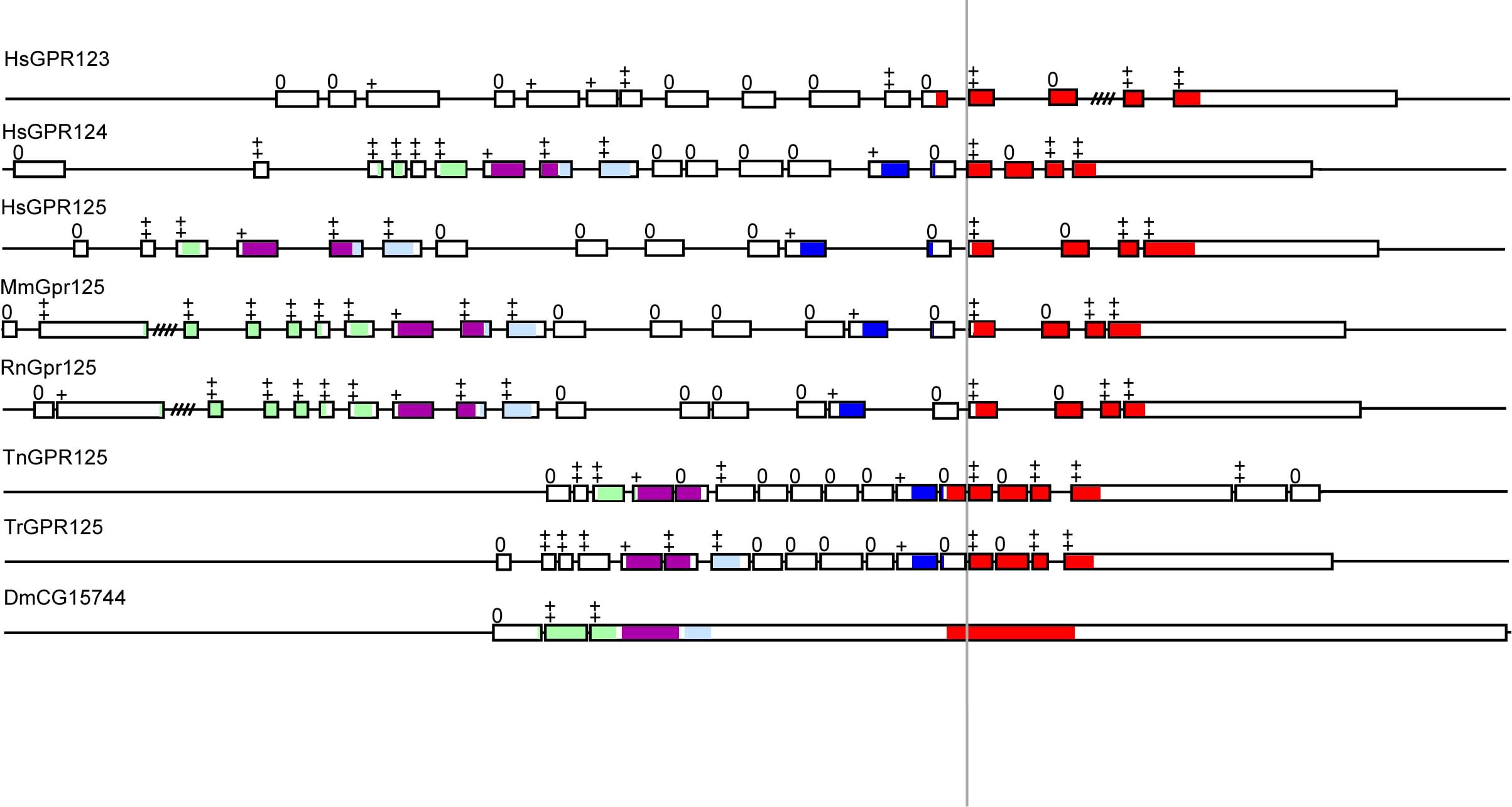

Supplement: Additional file 1 — Genomic structure of selected Adhesion GPCRs from human (Hs), mouse (Mm), rat (Rn), tetraodon (Tn), fugu (Tr) and drosophila (Dm) where exons are indicated by boxes and introns as lines. Domains were identified through the conserved domain database (rps-blast) against CDD -12589PSSMs with threshold value 0.1, and are depicted with different colours; Gal_Lectin (yellow), leucine-rich-repeats (green), immunoglobulins (purple), hormR – hormone binding domain (light blue), GPS – G protein-coupled receptor proteolytic site (blue), 7tm – transmembrane domain (red). Immunoglobulins consist of the domains IG and IGcam whereas leucine-rich-repeats consist of LRRCT, LRR_RI, LRR_TYP and COG4886. Exon-phases are displayed with 0 for zeroth phase, + for first phase and ++ for second phase. Interruptions in the sequence are indicated by //// for truncated intron sequence. The sequences have been aligned according to the second exon of the 7TM (see vertical line) since this location is present in all sequences. [file 1471-2202-9-97-S1.doc]

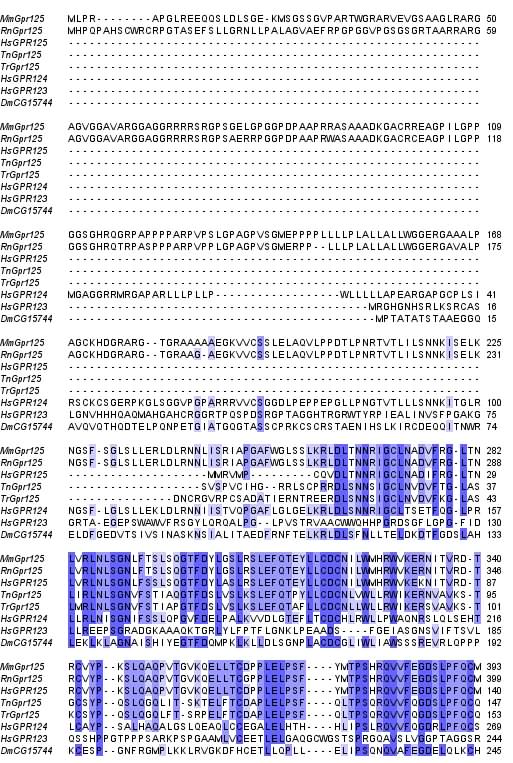


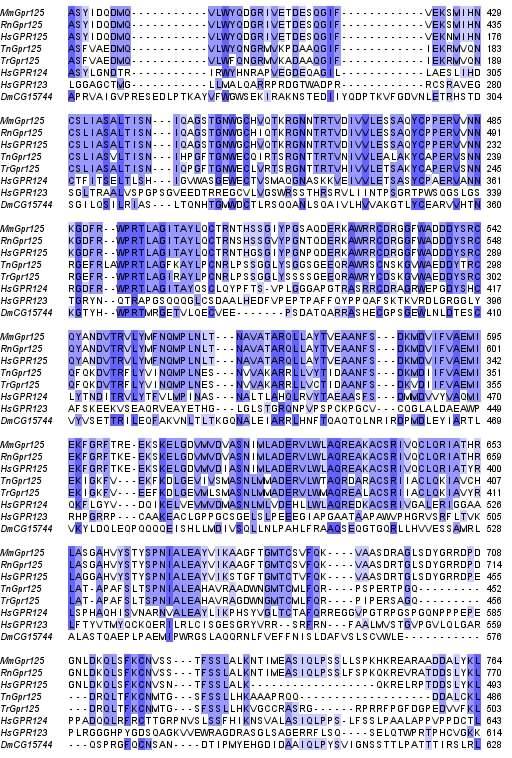


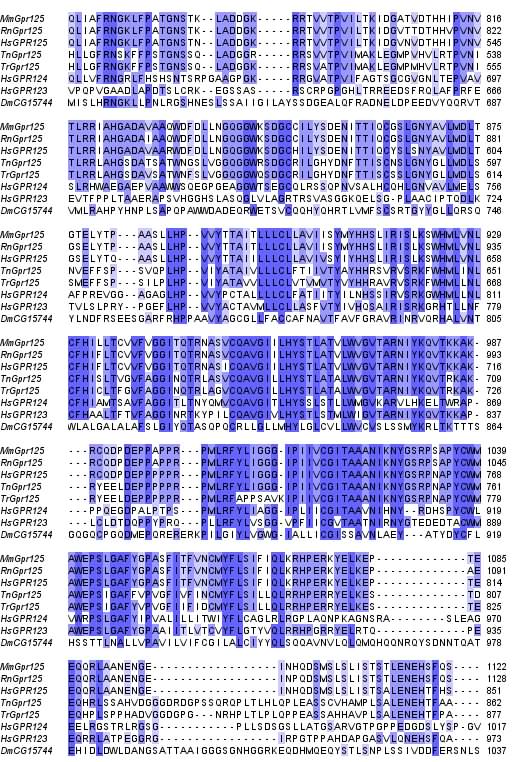


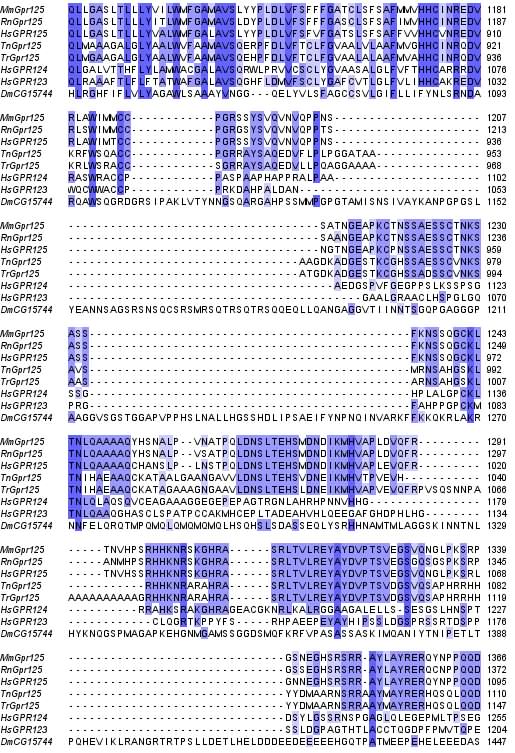


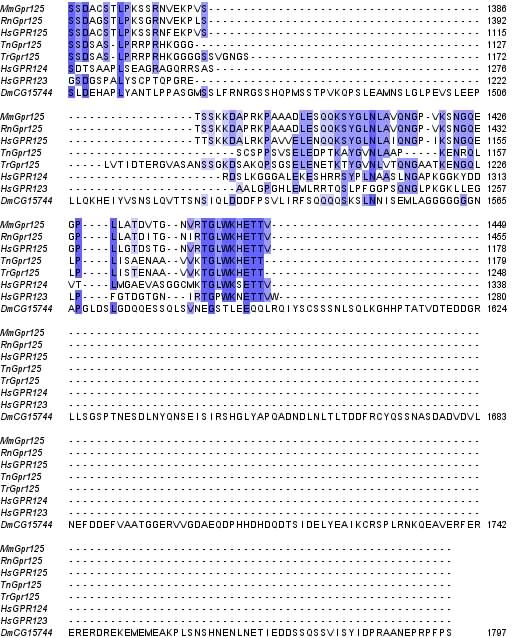

Supplement: Additional file 2 — Alignment from ClustalW 1.83 of human members of Adhesion Group III together with Gpr125-sequences from Mus musculus, Rattus norvegicus, Tetraodon nigroviridis, Takifugu rubripes and Drosophila melanogaster. The alignment has been edited in Jalview 2.2.1 and coloured according to percentage identity where darker colours indicate higher percentage identity. [file 1471-2202-9-97-S2.doc]

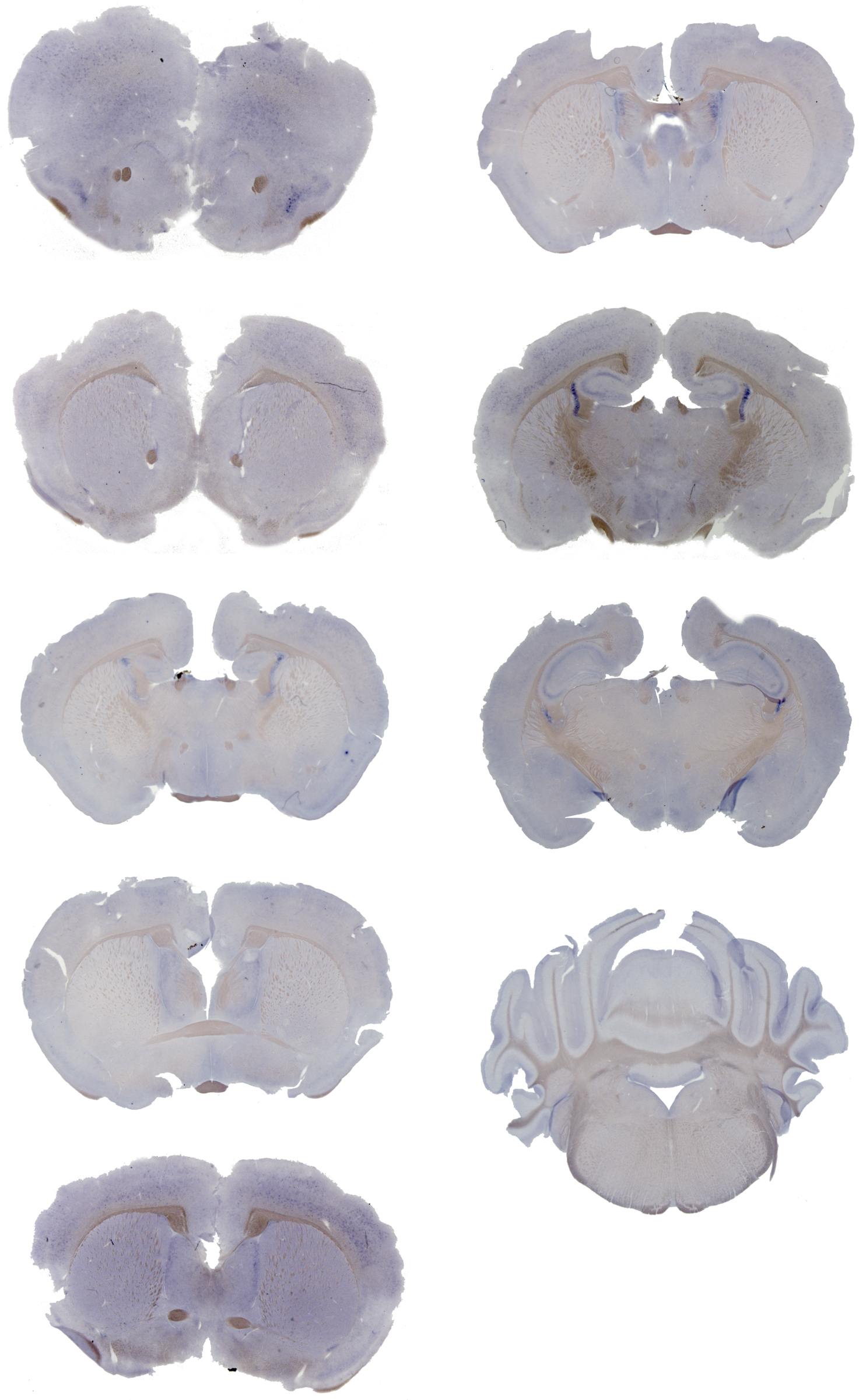

Supplement: Additional file 3 — In situ hybridization panel of GPR125 expression. In situ hybridization panel of GPR125 expression in the mouse brain on free floating sections using 400 ng of digoxigenin (DIG)-labeled mouse GPR125 antisense probe on coronal sections using BM-purple visible enzyme substrate. [file 1471-2202-9-97-S3.jpeg]

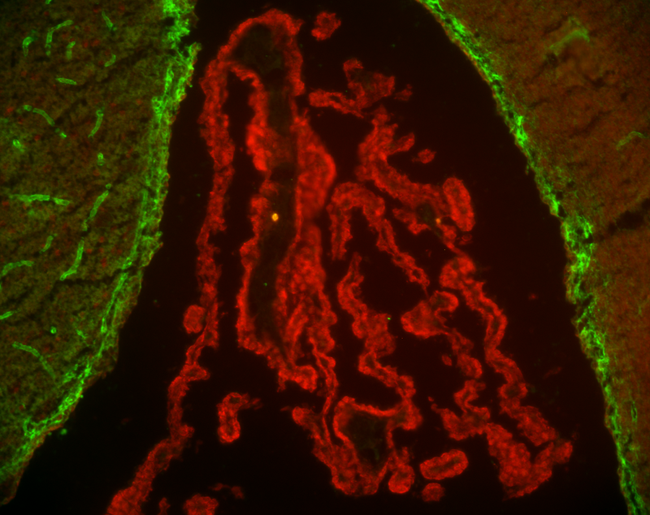

Supplement: Additional file 4 — GPR125 co-localization with vasculature. Labelling of the GPR125 protein (red) did not co-localize with monocarboxylate transporter MCT-2 (green) which is transporter found in the cerebral vasculature and the walls of the ventricle. [file 1471-2202-9-97-S4.tiff]
